# Confluence of Challenges: A Case Report of Superior Mesenteric Artery Syndrome Coexisting With Depressive Illness, Severe Weight Loss, and an Incidental Adnexal Cyst

**DOI:** 10.7759/cureus.57073

**Published:** 2024-03-27

**Authors:** Raamish A Khan, Zainab Khan, Zain B Shahid, Maryum Akhter, FNU Poombal

**Affiliations:** 1 Medicine, Bakhtawar Amin Medical and Dental College, Multan, PAK; 2 Medicine, Nishtar Medical University, Multan, PAK; 3 Surgery, Bakhtawar Amin Medical and Dental College, Multan, PAK; 4 Pathology, Nishtar Medical University, Multan, PAK

**Keywords:** small bowel obstruction, multidisciplinary approach, weight loss, depressive illness, superior mesenteric artery syndrome

## Abstract

In the case of a 24-year-old woman experiencing depressive illness, weight loss, vomiting, and hypoglycemia, initial suspicion of gastroenteritis shifted to reveal compensated metabolic acidosis and electrolyte imbalances. A subsequent CT scan revealed both superior mesenteric artery (SMA) syndrome and an incidental adnexal cyst, leading to treatment involving electrolyte correction and laparoscopic duodenojejunostomy, ultimately facilitating her recovery.

## Introduction

Superior mesenteric artery syndrome (SMAS) is an acquired vascular compression disorder in which acute angulation of the superior mesenteric artery (SMA) results in compression of the third part of the duodenum, leading to obstruction [1,2]. This condition is also referred to as Wilkie's syndrome. Research indicates that the prevalence of SMAS among the general population varies widely, ranging from 0.013% to 3.0% [3]. However, due to its underdiagnosis and nonspecific symptoms, the true prevalence might be higher. Additionally, SMAS carries a mortality rate of up to 33%, underscoring the importance of early recognition and intervention [4,5].

SMA syndrome originates from diverse factors, including a lean physique, heightened lumbar lordosis, visceral descent, and laxity in the abdominal wall. Notably, the reduction of mesenteric fat, accelerated by rapid and severe weight loss in conditions such as cancer, surgery, burns, trauma, or psychiatric disorders, is a pivotal contributor to its onset [6,7]. Typical indicators associated with SMA syndrome include feelings of nausea, bouts of vomiting, and upper central abdominal pain, coupled with manifestations of depression and significant weight loss in the affected individual, rendering it a less common occurrence [8].

In this article, we present a case study of a young woman experiencing severe weight loss, psychiatric illness, and metabolic acidosis, which led to the diagnosis of SMA syndrome.

## Case presentation

A 24-year-old woman with a one-year history of depressive illness due to familial issues was prescribed fluoxetine (an SSRI) and an anxiolytic. Over the past two months, she experienced significant weight loss, dropping from 50 kg to 30 kg, accompanied by a loss of appetite. She presented to the Emergency Department with vomiting occurring five times a day for three days, which contained food particles. Her blood pressure was 100/60, pulse rate was 100, and respiratory rate was 23 per minute.

In the ICU, she was drowsy and experienced two episodes of hypoglycemia. Initially suspected of having gastroenteritis, she underwent further evaluation, including a complete blood exam, arterial blood gas analysis, and renal parameter testing. Results revealed compensated metabolic acidosis (HCO_3_: 9.8 mEq/L) and electrolyte imbalance (hypokalemia and hypochloremia). Normal liver and renal function, mild anemia (Hb: 11.3 g/dL, Hct: 32.6%), and microcytic cells (lower MCV and MCH) were found. White blood cell and platelet counts were within normal ranges, indicating normal immune function and clotting ability.

Differential diagnosis included abdominal tuberculosis, malignancy, and autoimmune diseases. A CT scan confirmed SMA syndrome and incidentally detected an adnexal cyst.

**Figure 1:**
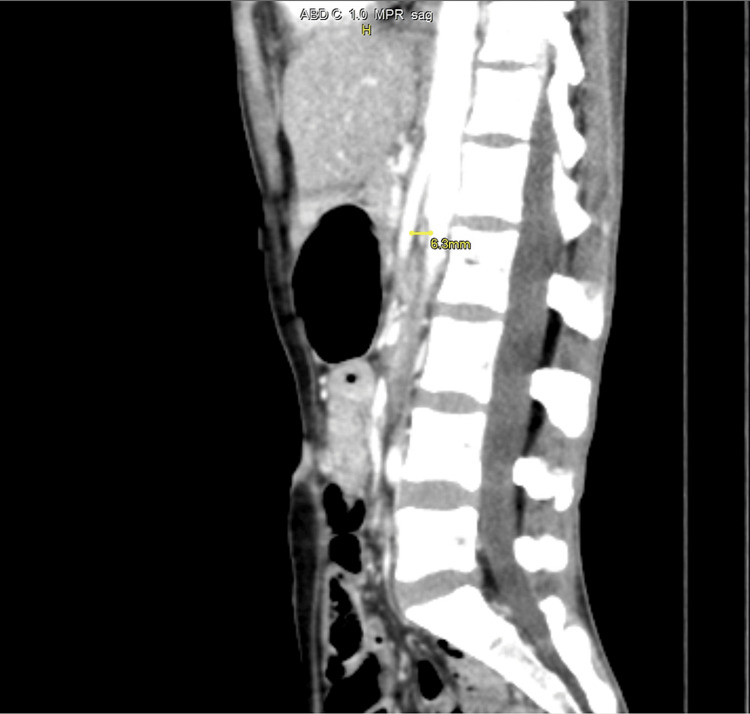
The image depicts an AOM distance measuring 6.3 mm. This is the sagittal section of the contrast-enhanced MRI of the abdomen. AOM, aortomesenteric

**Figure 2:**
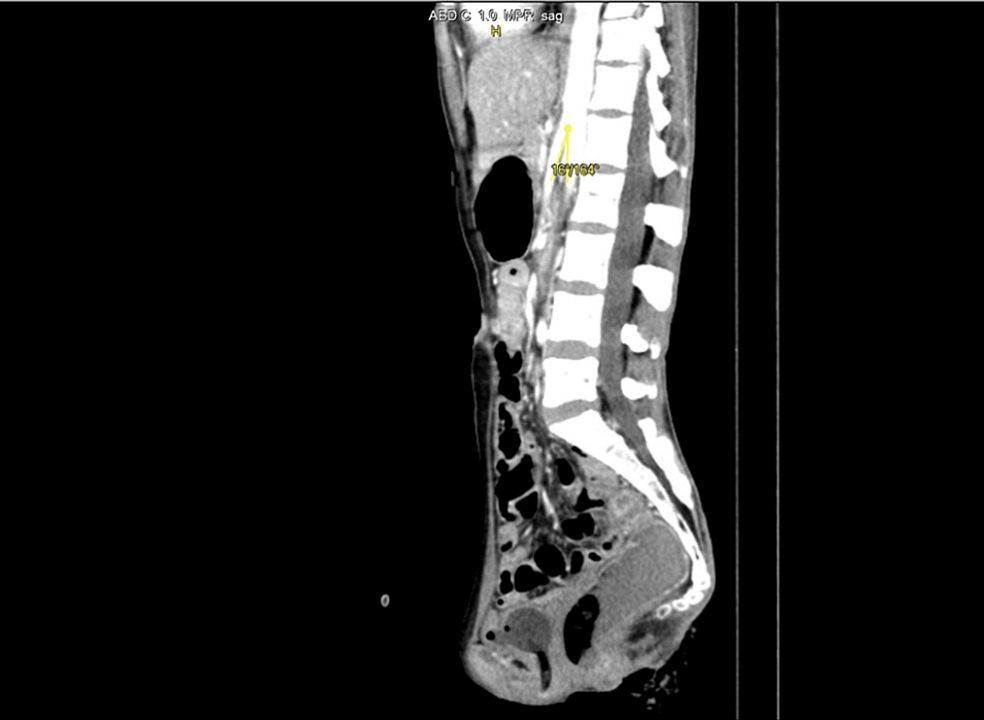
The image illustrates an AOM angle measuring 16º. This is the sagittal section of the contrast-enhanced MRI of the abdomen. AOM, aortomesenteric

**Figure 3:**
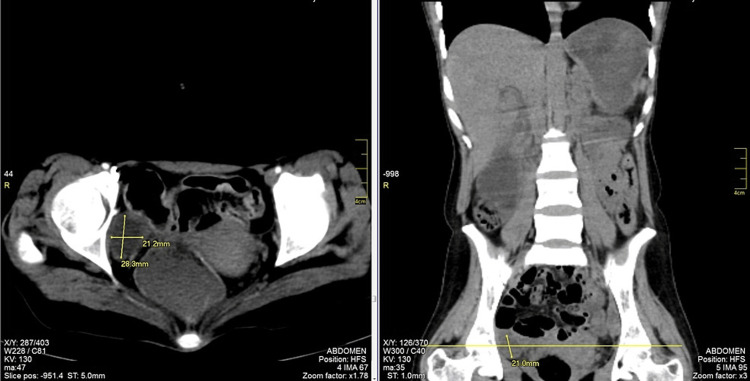
The CT scan revealed a right adnexal thin-walled cyst, measuring 2.8x2.1 cm. "HFS" in MRI stands for "Head First Supine," indicating a patient positioning with the head entering the scanner first while lying on their back. AOM, aortomesenteric

CT imaging findings were significant for diagnosing SMA syndrome, as evidenced by an aortomesenteric (AOM) angle of 16º and an AOM distance of 6.3 mm, values strongly suggestive of this condition. Further supporting this diagnosis was the observation of stomach and proximal duodenum distension, coupled with a collapse of the distal duodenum at the level of the SMA, as depicted in Figure 1 and Figure 2. Additionally, the CT scan revealed a right adnexal thin-walled cyst, measuring 2.8x2.1 cm (Figure 3), which is an incidental finding unrelated to the SMA syndrome. Furthermore, the imaging showed small bowel loops and the descending colon to be thick-walled and edematous, indicating the presence of an infective or inflammatory process.

The patient underwent management involving electrolyte replacement via two wide-bore intravenous lines, preparation for laparoscopic duodenojejunostomy, and anesthesia fitness. Despite a month-long attempt at conservative management, it proved ineffective. Subsequently, a duodenojejunostomy was planned, which the patient tolerated well. Intraoperatively, SMA compression was observed over the third part of the duodenum, along with proximal duodenal dilatation. The duodenojejunostomy was successfully performed. At the four-week follow-up post-surgery, the patient reported improvement in abdominal pain and distension.

A multidisciplinary approach, including input from a nutritionist for dietary planning, ongoing psychiatric support, and the continuation of antidepressant medication alongside psychotherapy, was advised.

## Discussion

Wilkie's syndrome, named after the doctor who studied its clinical and pathophysiological aspects in 64 patients, encompasses various terms such as chronic duodenal ileus, megaduodenum, compression of the AOM artery, obstruction of the arteriomesenteric duodenum, cast syndrome, and chronic duodenal pseudoobstruction [9].

Accurate diagnosis demands a heightened level of suspicion within the appropriate clinical context and necessitates a comprehensive radiological assessment [7]. To diagnose SMA syndrome, various imaging modalities such as CT scans, barium swallow, or contrast X-ray studies are employed. Contrast studies indicate duodenal obstruction, specifically impeding progression beyond the third part. CT imaging with vascular reconstruction enables the measurement of the AOM angle [10-12].

At the L1 vertebral level, the SMA emerges from the aorta's anterior side, descending with a sharp angle within the mesentery. Typically, the angle ranges from 38º to 65º, influenced by the mesenteric fat pad and correlating with body mass index. In normal conditions, the AOM distance is 10 mm to 28 mm [13,14]. In our case, the angle was reduced to 16º, and the aortomesenteric distance was reduced to 6.3 mm [13,14].

Haynes' criteria involve five aspects for diagnosing SMA syndrome via radiology: duodenal dilation, abrupt duodenal cutoff, counterflow, prolonged transit time, and obstruction relief with knee bending [15,16].

Therapeutic approaches for SMA syndrome encompass both conservative and surgical methods. The primary medical approach for SMA syndrome involves fluid resuscitation, total parenteral nutrition, the insertion of a nasoenteric tube beyond the obstruction for enteral feedings, as well as dietary modifications such as consuming small meals and adopting specific eating positions [17]. If conservative measures prove ineffective, surgical options should be contemplated [17].

Alongside gastric tube suction, intravenous metoclopramide can augment gastrointestinal motility, aiding in the process of decompression [18]. Surgical intervention is advisable when conservative treatments prove ineffective, particularly in elderly patients with a history of multiple abdominal operations, immobility (bed rest), prolonged SMA syndrome duration, and arteriosclerosis of SMA. Consideration of surgical therapy at an earlier stage is warranted to prevent the worsening of the patient's condition and the onset of complications [19]. Several surgical approaches can be employed to treat SMA syndrome, such as anterior duodenal transposition, gastroduodenostomy, gastrojejunostomy, duodenojejunostomy, Strong's procedure (ligament of Treitz division), duodenal lowering, Ladd's procedure, and endoscopic treatments involving lumen-apposing metal stents guided by EUS. The latter is considered a potential option based on case reports [17,18].

Since the inaugural laparoscopic duodenojejunostomy in 1998, the laparoscopic approach has become the favored choice among surgeons due to its proven safety and efficacy, boasting success rates ranging from 80% to 100% [17]. Additionally, the laparoscopic method has been associated with a reduction in the post-operative length of hospital stay [20].

Complications in SMA syndrome encompass mucosal injury reported in 25%-59% of patients. Mechanisms of sudden death, though unclear, are hypothesized to involve severe hypokalemia-induced arrhythmia. The compression of the inferior vena cava due to a dilated duodenum, along with alkalosis-induced pulmonary depression and increased abdominal pressure, can be inferred from published case studies and autopsies [18].

## Conclusions

This scenario emphasizes the importance of SMA syndrome as a potential factor in the differential diagnosis of unintentional weight loss and gastrointestinal issues, especially when dealing with mental health concerns. Early identification and a customized therapeutic strategy, combining medical and surgical approaches, are essential for obtaining positive results.

In conclusion, our comprehensive case report contributes to the understanding of the complex interplay between SMA syndrome, mental health, and incidental findings, emphasizing the need for a holistic approach to patient care.
